# Acute heroin intoxication in a baby chronically exposed to cocaine and heroin: a case report

**DOI:** 10.1186/1752-1947-5-288

**Published:** 2011-07-05

**Authors:** Xavier Joya, Bibiana Fríguls, Marta Simó, Ester Civit, Rafael de la Torre, Antonio Palomeque, Oriol Vall, Simona Pichini, Oscar Garcia-Algar

**Affiliations:** 1 Unitat de Recerca Infancia i Entorn (URIE), Institut Municipal d'Investigació Mèdica (IMIM)-Hospital del Mar, Barcelona, Spain; 2 Departament de Pediatria, Hospital San Joan de Déu, Barcelona, Spain; 3 Unitat de Recerca en Farmacologia Humana i Neurociències, Institut Municipal d'Investigació Mèdica-Hospital del Mar, Barcelona, Spain; 4 Department of Therapeutic Research and Medicines Evaluation, Istituto Superiore di Sanità, Rome, Italy

## Abstract

**Introduction:**

Acute intoxication with drugs of abuse in children is often only the tip of the iceberg, actually hiding chronic exposure. Analysis using non-conventional matrices such as hair can provide long-term information about exposure to recreational drugs.

**Case presentation:**

We report the case of a one-month-old Caucasian boy admitted to our pediatric emergency unit with respiratory distress and neurological abnormalities. A routine urine test was positive for opiates, suggesting an acute opiate ingestion. No other drugs of misuse, such as cocaine, cannabis, amphetamines or derivatives, were detected in the baby's urine. Subsequently, hair samples from the baby and the parents were collected to evaluate the possibility of chronic exposure to drug misuse by segmental analysis. Opiates and cocaine metabolites were detected in hair samples from the baby boy and his parents.

**Conclusions:**

In light of these and previous results, we recommend hair analysis in babies and children from risky environments to detect exposure to heroin and other drug misuse, which could provide the basis for specific social and health interventions.

## Introduction

During the past two decades, there has been a substantial increase in illicit drug consumption in Europe, particularly in Mediterranean areas such as Spain [1]. It has been proven repeatedly that questionnaires with respect to drug use are far from being accurate [2]. For this reason, in addition to questionnaires, it is advisable to use an objective biological marker that maintains its sensitivity for at least a few days after the end of the exposure and that may yield a cumulative picture of repeated exposure to drug misuse. Hair, a well established matrix for this purpose, allows a relatively long retrospective identification of a large number of substances that usually disappear quickly from blood and urine [3].

Hair testing is especially useful in the case of newborns and children for the assessment of both pre-natal and post-natal exposure to drug misuse [4,5]. The rationale for the approach of this manuscript is the need for objective assessment of long-term exposure in child custody cases, possible parent prosecution for exposing children passively or actively to drug misuse, and for the disclosure of repeated exposure to cocaine in cases of severe acute intoxication with cocaine alone or together with other illicit drugs. Hair is a biological matrix that permits a relatively long retrospective identification of substances; this long window of detection includes months to years, and the growth rate according to the Society of Hair Testing is 1 cm per month.

## Case presentation

A one-month-old Caucasian breastfed baby was admitted to the emergency department (ED) with respiratory distress. The parents mentioned that the baby showed superficial breathing with pauses during the past hour. On physical examination, our patient presented with generalized cyanosis, fixed and constricted pupils, muscular hypotony and respiratory failure. The mother admitted consumption of cannabis and beer the night before followed by breastfeeding of the baby afterward. A blood cell count and serum biochemistry were unremarkable, but the venous gasometrical results showed respiratory acidosis. At that point, the mother mentioned the possible ingestion of acetaminophen-codeine tablets. A screening for principal misused drugs (opiates, cocaine, cannabis, amphetamine) in urine was performed by a cloned enzyme donor immunoassay (CEDIA; Microgenics, Barcelona, Spain), and a positive result for opiates was obtained. Subsequently, a gas chromatography-mass spectrometry (GC-MS) urine analysis [6] confirmed the presence of heroin metabolites (free and conjugate morphine for a total of 312 ng/mL and codeine at 26 ng/mL).

In the ED, our patient experienced a not-limited breathing pause, so he was intubated and mechanically ventilated. He was transferred to the pediatric intensive care unit with fentanyl infusion. Analysis of his cerebrospinal fluid was normal, and central nervous system culture results were negative. Also, nasopharyngeal swab results were negative for common respiratory viruses. An ultrasound examination of the brain through the anterior fontanel was unremarkable, and echocardiography showed only a patent oval foramen. After 48 hours, our patient had correct respiratory response, and mechanical ventilation was discontinued. Our patient recovered completely without any neurological impairment and was discharged from the hospital and held by the authorities to keep him away from his parents. There was a strong suspicion that our patient had been in an environment of drug misuse by the mother or by other caregivers. To verify the suspicion, a GC-MS hair analysis for our patient and his parents was suggested. The parents agreed to the analysis, and hair was cut from the vertex region of the scalp.

All three hair samples (12 cm from the mother, 3 cm from the father and 2 cm from our patient) were analyzed for cocaine, benzoylecgonine, δ-9-tetrahydrocannabinol, 6-monoacetylmorphine, morphine and codeine [7]. In addition, the mother's hair was also submitted to segmental analysis corresponding approximately to the lifetime of the child, including pre-natal life. For the segmental analysis, the hair sample was divided into four segments of 3 cm each. After a standardized washing procedure, the samples were tested using a GC-MS assay [7]. The mother's hair appeared unbleached and not treated with any aggressive cosmetics such as straighteners. The results of the hair testing suggested parental cocaine and heroin consumption and a likely pre-natal and post-natal exposure of the baby to these drugs (Table 1). Our patient and his father had higher concentrations of the principal metabolite, 6-monoacetylmorphine (6-MAM), than the mother's hair.

**Table 1:** Toxicological findings in hair from our patient and his parents

Specimens	Length of hair (cm)	Cocaine (ng/mg)	Benzoylecgonine (ng/mg)	Morphine (ng/mg)	6-MAM (ng/mg)	Codeine (ng/mg)
Patient's hair	2	17.5	2.2	2.4	8.1	0.4

Father's hair	3	11.8	1.7	6.4	8.0	1.8

Mother's hair	12 (total)					

	0-3	2.5	1.6	0.4	0.8	0.1

	3-6	3.7	2.1	0.2	0.6	0.1

	6-9	4.4	4.3	0.2	0.7	0.05

	9-12	3.0	5.5	0.3	0.7	0.1

6-MAM = 6-monoacetylmorphine.

## Discussion

To the best of our knowledge, this is the first case reported in the literature describing heroin intoxication in a baby chronically exposed to heroin and cocaine, probably caused by the combined effect of chronic exposure through the placenta (pre-natal), breast milk and skin (post-natal) and acute exposure through smoke and breast milk.

The results of our patient's urine test revealed acute heroin intoxication. The mother's declaration regarding the possible ingestion of acetaminophen-codeine by our patient proved to be false because urine analysis revealed the main presence of free and conjugated morphine and a lower proportion of codeine. This first result suggests an acute intoxication through breast milk or passive exposure by environmental inhalation of smoked heroin. The mother refused breast milk sampling and denied personal use of opiates and cocaine, as well as the contact of the child with other drug users or the presence of illicit substances in the household.

A second relevant result in this case was that hair testing disclosed parental repeated consumption of heroin (indeed, its principal metabolite, 6-MAM, was always identified in all the hair samples) and cocaine and the subsequent repeated passive exposure of our patient to these two misused drugs.

Because our patient was only 1 month old, the high concentration of opiates and cocaine in his hair could not be attributed unequivocally to pre-natal or post-natal exposure to these misused drugs. These concentrations could be explained by any of these scenarios: permanent feto-placental contact with a drug-consuming mother during intra-uterine life, passive inhalation or forced drug misuse, or by our patient putting contaminated objects in his mouth [8,9]. In fact, the source and exact timing of exposure are of little consequence; the case report shows that repeated exposure of our patient to cocaine and heroine was objectively highlighted by testing in two different biological matrices accounting for different time windows of exposure [10].

In Spain, heroin and cocaine are usually consumed together by smoking, and it must be recommended not to use them in areas where newborns and babies may reside or be present.

## Conclusions

In contrast to drug testing in conventional matrices, which can account for acute exposure, testing in non-conventional matrices can shed light on past and possibly repeated exposure and can disclose the possibility of a baby living in an unsafe and high-risk environment in which exposure to drug misuse takes place. The accurate assessment of both acute and repeated exposure of a child to drug misuse by the use of objective biomarkers is of major importance because it provides the basis for appropriate immediate treatment, adequate medical follow-up and social intervention. Finally, in cases of acute exposure to drug misuse, we suggest further investigation of the possibility of chronic exposure as well.

## Consent

Written informed consent was obtained from our patient's next-of-kin for publication of this case report and any accompanying images. A copy of the written consent is available for review by the Editor-in-Chief of this journal.

## Competing interests

The authors declare that they have no competing interests. The authors alone are responsible for the content and writing of this paper.

## Authors' contributions

XJ analyzed patient data, reviewed the literature and was a major contributor to writing the manuscript. BF analyzed patient data and was a major contributor to writing the manuscript. MS was the clinician in charge of the child and contributed to writing the manuscript. EC was an important laboratory technician in biomarkers analyses and contributed to writing the manuscript. RT was the head of the laboratory responsible and contributed to writing the manuscript. AP was the other clinician in charge of our patient and contributed to writing the manuscript. OV was the pediatrician responsible for coordination of data and sample flow and contributed to writing the manuscript. SP was the major expert in laboratory analysis of biomarkers in alternative matrices and contributed to writing the manuscript. OGA analyzed patient data, reviewed the literature and the final manuscript and was the main contributor to writing the manuscript. All authors read and approved the final manuscript.
